# Sexual dysfunction and quality of life in males with genital warts

**DOI:** 10.1186/s12610-025-00274-1

**Published:** 2025-06-19

**Authors:** Mohamad M. Elhamady, Rabie B. Atallah, Osama A. Hashem, Mahmoud A. Abdelaty, Mohamed L. Elsaie

**Affiliations:** 1 Department of Dermatology, Venereology and Andrology, Damietta Faculty of Medicine, Al-Azhar University, Damietta, Egypt; 2 Department of Public Health and Community Medicine, Damietta Faculty of Medicine, Al-Azhar University, Damietta, Egypt; 3 Department of Dermatology, National Research Centre , Medical Research and Clinical Studies Institute, Giza, Egypt

**Keywords:** Genital warts, Sexual dysfunction, Quality of life, Human papilloma virus, Condylomes génitauw, Dysfonction sexuelle, Qualité de Vie, Virus du Papillome humain

## Abstract

**Background:**

Genital warts caused by human papillomavirus, significantly impact male sexual function and quality of life. However, the extent of dysfunction varies across studies, necessitating further investigation. This study aims to assess sexual dysfunction and quality of life in men with GWs.

**Results:**

A cross-sectional study was conducted among men diagnosed with GWs. Sexual function was evaluated using the International Index of Erectile Function while the Dermatology Life Quality Index measured quality-of-life impairment. Data were analyzed to determine the severity and distribution of dysfunction across domains. Erectile dysfunction was prevalent, with a mean International Index of Erectile Function score of 14.3 ± 4.2, indicating moderate dysfunction. Orgasmic satisfaction (5.8 ± 2.1) and overall satisfaction (6.5 ± 2.4) were also moderately affected. Quality of life was significantly impaired, with 36.7% experiencing moderate impact, 26.7% reporting a very large impact, and 8% suffering an extreme impact.

**Conclusion:**

Genital warts contribute to substantial sexual dysfunction and diminished quality of life. The results highlight the need for routine sexual health assessments and psychological support. Integrating medical and psychosocial care may improve patient outcomes.

**Supplementary Information:**

The online version contains supplementary material available at 10.1186/s12610-025-00274-1.

## Résumé

**Contexte:**

Les condylomes génitaux causés par le virus du papillome humain ont un impact significatif sur la fonction sexuelle masculine et sur la qualité de vie. Cependant, l’étendue de la  dysfonction varie d’une étude à l’autre, ce qui nécessite des investigations plus approfondies. Cette étude vise à évaluer la dysfonction sexuelle et la qualité de vie des hommes atteints de condylomes génitaux (CG).

**Résultats:**

Une étude transversale a été menée auprès d’hommes ayant reçu un diagnostic de CG. La fonction sexuelle a été évaluée à l’aide de l’indice international de la fonction érectile, tandis que l’indice de qualité de vie dermatologique a mesuré l’altération de la qualité de vie. Les données ont été analysées pour déterminer la gravité et la répartition de la dysfonction entre les domaines.

La dysfonction érectile était prévalente, avec un score moyen de l’indice international de la fonction érectile de 14,3 ± 4,2, indiquant une dysfonction modérée. La satisfaction orgasmique (5,8 ± 2,1) et la satisfaction globale (6,5 ± 2,4) ont également été modérément affectées. La qualité de vie a été considérablement altérée, 36,7 % ayant subi un impact modéré, 26,7 % ayant signalé un impact très important et 8 % ayant subi un impact extrême.

**Conclusions:**

Les condylomes génitaux contribuent à une dysfonction sexuelle importante et à une diminution de la qualité de vie. Les résultats soulignent la nécessité d’évaluations régulières de la santé sexuelle et d’un soutien psychologique. L’intégration de soins médicaux et psychosociaux peut améliorer les résultats pour les patients

**Graphical Abstract:**

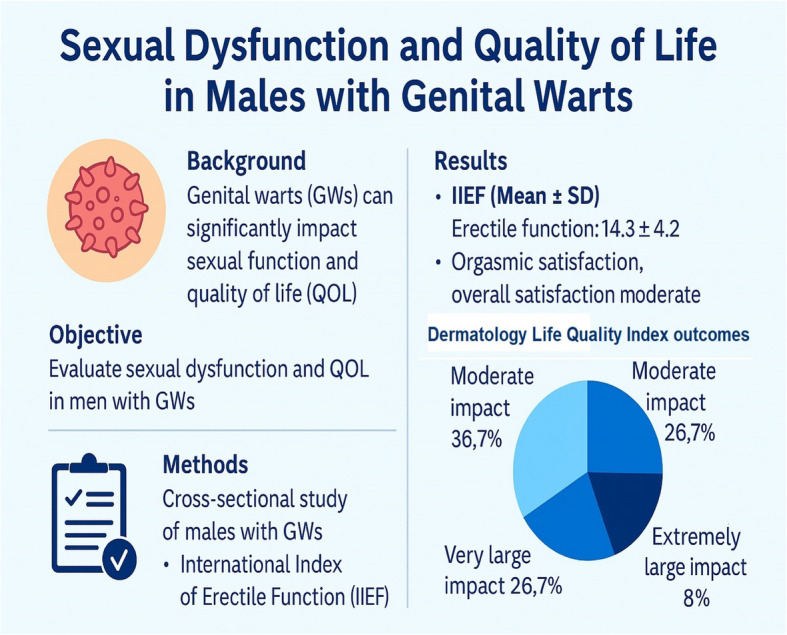

## Introduction

Genital warts (GWs), caused predominantly by low-risk human papillomavirus (HPV) types 6 and 11, represent one of the most common sexually transmitted infections (STIs) globally, with a considerable burden on public health and individual well-being. While much attention has been given to their clinical management and prevention, the broader psychosocial and sexual consequences of GWs—particularly in men—have received comparatively limited focus. Beyond the visible lesions, men with GWs often experience profound psychological distress, feelings of shame, and sexual dysfunction, which are increasingly documented in relation to erectile dysfunction [1, 2], which collectively diminish quality of life and affect interpersonal relationships [3].

The pathophysiological link between HPV infection and sexual dysfunction remains complex and multifactorial. Chronic inflammation, psychological comorbidity, and the stigma associated with visible genital lesions may all contribute to impaired sexual function, as shown in recent studies linking HPV to vasculitis, inflammation, and depression [4–7]. Recent literature suggests that men with GWs are at increased risk for erectile dysfunction, reduced sexual satisfaction, and diminished self-esteem, yet the extent and nature of these impairments vary considerably across populations and settings [8].

Despite these observations, standardized assessments of sexual function and quality of life in men with GWs remain sparse, especially in resource-limited contexts. Most existing studies focus on female populations or utilize general health metrics that may overlook domain-specific dysfunctions. There is a critical need for validated, multidimensional tools that accurately capture the sexual and psychosocial burden of GWs in men [9].

This study aimed to assess the prevalence and severity of sexual dysfunction and quality-of-life impairment in men diagnosed with genital warts using two widely recognized instruments: the International Index of Erectile Function (IIEF) and the Dermatology Life Quality Index (DLQI). By identifying the key clinical and behavioral factors associated with these outcomes, this research seeks to inform a more holistic approach to the management of men with genital warts, emphasizing not only lesion treatment but also sexual health and psychosocial support.

### Patients and methods

This study was conducted as a cross-sectional analysis. The research was carried out at the outpatient clinic of the Dermatology, Venereology, and Andrology Department, Al-Azhar University Hospital in New Damietta, Egypt between July 2024 and January 2025.Sampling and Sample Size Calculation: Participants were enrolled consecutively from dermatology clinic attendees. The sample size was calculated based on the prevalence of sexual dysfunction among males with genital warts reported in prior research [8]. Using Epi Info version 7.2.4.0, with an expected prevalence of 10%, a 95% confidence level, and a margin of error of 5%, the required sample size was determined to be 150 male participants.

#### Inclusion criteria

Participants eligible for inclusion in the study were adult males, aged 18 years or older, who had been clinically diagnosed with genital warts for a minimum of two months and had engaged in sexual activity within the preceding four weeks.

Individuals were excluded if they had any medical or psychiatric condition known to affect male sexual function, including diabetes, hypertension, autoimmune or cardiovascular diseases. Patients were also excluded if they were taking medications that could influence erectile or sexual function, such as antihypertensives, antidepressants, blood sugar regulators, or amphetamines.

#### Informed consent

Written informed consent was obtained from all participants prior to their inclusion in the study. The consent form detailed the study's purpose.

#### Ethical considerations

The study protocol was reviewed and approved by the local ethics committee and the institutional research board (IRB) at the Faculty of Medicine, Al-Azhar University, Damietta. Confidentiality and personal privacy were strictly maintained throughout all stages of the study. Data collected were used solely for research purposes.

## Methods

A comprehensive medical history was obtained from each participant. Demographic information such as age, place of residence, educational attainment, and employment status was recorded. Marital history included the duration of marriage, as well as the spouse’s age, educational background, and occupation. In addition, a detailed account of the presenting illness was documented, including the onset, duration, and progression of genital warts. Participants were also asked about any prior medical conditions, both systemic and dermatological.

Each participant underwent a general physical examination to rule out systemic illnesses. A thorough dermatological assessment was conducted to evaluate the number, location, and size of genital warts, along with the presence or absence of lesions at distant body sites.

### Assessment of male sexual function

Sexual function was measured using the 15-item IIEF questionnaire, which covers five key domains and uses Likert-type scales for scoring. It evaluates five domains: erection, orgasm, desire, intercourse satisfaction, and overall satisfaction. Scores ranged from 0 to 75, with higher scores indicating better sexual function. The status of patients’ erectile function was also considered as follows: 0–10 (severe erectile dysfunction (ED), 11–16 (moderate ED), 17–21 (moderate to mild ED), 22–25 (mild ED), 26–30 (no ED) [9].

### Assessment of Quality of Life (QoL)

We used the validated Arabic version of the Dermatology Life Quality Index (DLQI to evaluate impact of genital warts on QoL [10]. This validated tool consists of 10 items addressing symptoms, feelings, daily activities, leisure, work, school, personal relationships, and treatment. Each item is scored on a scale from 0 to 3, with total scores ranging from 0 (no impairment) to 30 (maximum impairment). QoL impact was classified as:0–1: No effect; 2–5: Small effect; 6–10: Moderate effect; 11–20: Very large effect; while 21–30: Extremely large effect.

### Statistical analysis

Data were analyzed using SPSS version 22. Categorical variables were described as frequencies and percentages, and continuous variables as means ± standard deviation or medians with ranges based on distribution. The Kolmogorov–Smirnov test was applied to assess normality of continuous data. For between-group comparisons, the Chi-square test was used for categorical variables, the student’s t-test for normally distributed continuous variables, and the Mann–Whitney U test for non-normally distributed data. Pearson correlation coefficients were calculated to evaluate associations between continuous variables. Multivariate linear regression analyses were conducted to identify independent predictors of Dermatology Life Quality Index (DLQI) and International Index of Erectile Function (IIEF) scores. The variables included in the regression models were: duration of genital warts, size of warts, number of warts, smoking status, condom use, age group, employment status, and educational level. These variables were selected based on their established or plausible influence on sexual function and quality of life, as supported by existing literature and clinical rationale.

## Results

### Demographic characteristics

This cross-sectional study included 150 adult men diagnosed with genital warts at the Dermatology, Venereology, and Andrology outpatient clinic of Al-Azhar University Hospital, New Damietta, Egypt. The mean age of participants was 28.3 years (SD ± 7.2), with the largest proportion (40%) falling within the 25–29 age group. The distribution of education levels showed that 40% of participants held a university degree, while 7.3% had no formal education. The majority (74%) were employed, and 62% resided in rural areas (Table 1).

**Table 1:** Demographic characteristics of the patients and clinical features of genital warts

Characteristic	Category	n (%)
Age (Mean ± SD)	28.3 ± 7.2	
Age Group (years)	18–24	35 (23.3)
	25–29	60 (40.0)
	30–39	40 (26.7)
	≥ 40	15 (10.0)
Education Level	Not Educated	11 (7.3)
	Below High School	14 (9.4)
	High School Graduate	65 (43.3)
	University Degree	60 (40.0)
Employment Status	Employed	111 (74.0)
	Unemployed	39 (26.0)
Residency	Urban	57 (38.0)
	Rural	93 (62,0)
Number of Warts	Single	18 (12)
	Multiple	132 (88)
Size of Warts (cm)	< 1	74 (49.3)
	1–2	56 (37.3)
	> 2	20 (13.3)
Duration of Warts (months)	≤ 6	95 (63.3)
	> 6	55 (36.7)

Presents demographic and clinical characteristics of genital wart patients. Includes age, education, and wart attributes

*DLQI* Dermatology Life Quality Index, *n (%)* number; (percentage)

### Clinical features of genital warts

Among the studied patients, 88% had multiple warts, while 12% had a single wart. Nearly half (49.3%) of the warts were smaller than 1 cm, whereas 37.3% measured 1–2 cm, and 13.3% exceeded 2 cm in size. The duration of warts was six months or less in 63.3% of cases, while 36.7% had warts persisting for more than six months (Table 1).

### Sexual function assessment

The International Index of Erectile Function (IIEF) scores categorized sexual dysfunction severity among participants. Mild dysfunction was the most prevalent (29.3%), followed by mild-to-no dysfunction (24%). Severe dysfunction was observed in 10.7% of participants, while 20.7% reported no dysfunction (Table 2). Domain-wise analysis revealed moderate erectile dysfunction (mean = 14.3 ± 4.2), reduced orgasmic function (mean = 5.8 ± 2.1), and moderate sexual satisfaction (mean = 8.2 ± 3.0) and a moderate overall satisfaction (6.5 ± 2.4) Table 3.

**Table 2:** Categorization of patients based on cumulative scores of International Index of Erectile Function (IIEF)

Cumulative Score Range	Category	Number of Patients (n)	Percentage (%)
0–15	Severe dysfunction	16	10.70%
16–30	Moderate dysfunction	23	15.30%
31–45	Mild dysfunction	44	29.30%
46–60	Mild-to-no dysfunction	36	24.00%
61–75	No dysfunction	31	20.70%
Total		150	100.00%

Categorizes patients by severity of erectile dysfunction using IIEF scores. Severity ranges from severe to no dysfunction

*IIEF* International Index of Erectile Function

**Table 3:** Sexual function assessment domains based on International Index of Erectile Function (IIEF)

Domain	Mean ± SD	Range	Interpretation
Erectile Function	14.3 ± 4.2	5–20	Moderate dysfunction
Orgasmic Function	5.8 ± 2.1	1–10	Reduced satisfaction
Sexual Desire	6.9 ± 2.3	2–10	Moderate desire
Intercourse Satisfaction	8.2 ± 3.0	3–12	Moderate satisfaction
Overall Satisfaction	6.5 ± 2.4	2–9	Moderate overall satisfaction

Displays domain-specific sexual function scores from IIEF. Shows dysfunction levels in erection, desire, satisfaction

*IIEF* International Index of Erectile Function

### Impact on quality of life

The Dermatology Life Quality Index (DLQI) scores demonstrated that 36.7% of patients experienced a moderate impact on life, while 26.7% reported a very large impact. Only 5.3% of patients experienced no impact (Supplementary Table 1). All domains of sexual function measured by the IIEF show a negative correlation with DLQI, meaning that worse quality of life is associated with poorer sexual function. Erectile function and intercourse satisfaction had the strongest correlations with DLQI. All correlations were statistically significant (*p* < 0.05) (Table 4).

**Table 4:** Correlation between DLQI score and IIEF domains

IIEF Domain	Mean DLQI Score	Correlation Coefficient(r)	*p-*value
Erectile Function	12.8 ± 4.1	−0.47	0.001
Orgasmic Function	11.6 ± 3.9	−0.32	0.01
Sexual Desire	10.9 ± 4.2	−0.28	0.03
Intercourse Satisfaction	10.83 ± 3.7	−0.42	0.001
Overall Satisfaction	11.2 ± 4.0	−0.36	0.005

Shows correlation between quality of life and sexual function. Negative correlation values indicate greater impairment

*DLQI* Dermatology Life Quality Index, *IIEF* International Index of Erectile Function, *r* correlation coefficient

### Influence of smoking, wart duration, and condom use on sexual dysfunction

Smoking status significantly affected sexual dysfunction prevalence. Among smokers (74% of the sample), severe dysfunction was reported in 7.3%, compared to 3.3% in non-smokers (Supplementary Table 2). Patients with warts persisting longer than six months had higher rates of severe dysfunction (20%) than those with shorter durations (5.3%) (Supplementary Table 3). Condom use also influenced quality of life, with those who always used condoms reporting lower DLQI scores and lower rates of severe dysfunction compared to inconsistent or non-users (Supplementary tables 4–5).

### Employment status and sexual dysfunction

Employment status significantly influenced DLQI scores, with employed individuals more likely to report no impact or small impact compared to unemployed individuals, who had higher proportions of moderate-to-extremely large impact (Table 5). Age group analysis suggested that severe dysfunction was most common in the 18–24 and 25–29 age groups, while no dysfunction was most prevalent in the 30–39 age group (Supplementary Table 6).

**Table 5:** Correlation between employment status and DLQI scores

DLQI Score Range	Impact on Quality of Life	Employed n (%)	Unemployed n (%)	*p-*value
0–1	No impact	15 (13.50)	3 (7.70)	0.001
2–5	Small impact	40 (36.0)	10 (25.60)	0.001
6–10	Moderate impact	35 (31.50)	15 (38.50)	0.001
11–20	Very large impact	15 (13.50)	7 (17.90)	0.05
21–30	Extremely large impact	6 (5.40)	4 (10.30)	0.06
Total		111 (100)	39 (100)	

Compares DLQI scores based on employment status. Chi-square test applied to determine significance

*DLQI * Dermatology Life Quality Index

*p* value significant at < 0.05

### Multivariate analysis of factors affecting DLQI and IIEF scores

The multivariate linear regression model for DLQI explained 42.1% of the variance in quality-of-life scores (adjusted R^2^ = 0.421), while the model for IIEF scores explained 37.8% of the variance in sexual function (adjusted R^2^ = 0.378). Duration and size of warts (β = −0.48, *p* = 0.001), smoking status (β = −0.36, *p* = 0.004), and number of warts (β = −0.3, *p* = 0.01) as significant predictors of DLQI impairment. The addition of wart size as a variable showed a statistically significant impact on both DLQI and IIEF scores. Similarly, duration of warts (β = −0.35, *p* = 0.003), smoking (β = −0.3, *p* = 0.005), and number of warts (β = −0.28, *p* = 0.02) were significantly associated with lower IIEF scores (Tables 6 and 7). Employment status and residency did not show statistically significant effects on these outcomes.

**Table 6:** Multivariate analysis of factors affecting DLQI scores

Factors	Coefficient (β)	*p-*value	Interpretation
Duration of Warts	−0.48	0.001	Significant negative impact on DLQI
Size of Warts	−0.33	0.008	Moderate negative impact on DLQI
Smoking Status	−0.36	0.004	Moderate negative impact on DLQI
Condom Use	−0.25	0.02	Mild negative impact on DLQI
Employment Status	0.12	0.1	Not statistically significant
Residency	0.18	0.15	Not statistically significant
Education Level	−0.2	0.03	Mild negative impact on DLQI
Age Group	−0.15	0.05	Borderline statistical significance
Number of Warts	−0.3	0.01	Significant negative impact on DLQI

Identifies predictors of DLQI scores via regression. Factors include wart size, duration, smoking

*DLQI* Dermatology Life Quality Index, *β* regression coefficient

*p* value significant at < 0.05

**Table 7:** Multivariate analysis of factors affecting IIEF scores

Factors	Coefficient (β)	*p-*value	Interpretation
Duration of Warts	−0.35	0.003	Significant negative impact on IIEF
Size of warts	−0.29	0.01	Moderate negative impact on IIEF
Smoking Status	−0.3	0.005	Moderate negative impact on IIEF
Condom Use	−0.22	0.015	Mild negative impact on IIEF
Employment Status	0.08	0.12	Not statistically significant
Residency	0.1	0.2	Not statistically significant
Education Level	−0.18	0.04	Mild negative impact on IIEF
Age Group	−0.12	0.06	Borderline statistical significance
Number of Warts	−0.28	0.02	Moderate negative impact on IIEF

Regression model for erectile function using IIEF. Predictors include wart-related and demographic variables

*IIEF* International Index of Erectile Function, *β* regression coefficient

*p* value significant at < 0.05

## Discussion

Genital warts (GWs), primarily caused by the human papillomavirus (HPV), are one of the most common sexually transmitted infections worldwide. Though there is abundant documentation in literature regarding their physical manifestations and effects; their psychosocial, sexual and other implications, particularly in males still remain to be elucidated. Our study aimed to discover the impact of GWs on male sexual function and quality of life [3].

### Prevalence and domains of sexual dysfunction

In our cohort of 150 men with GWs, a substantial proportion exhibited varying degrees of sexual dysfunction. Specifically, 10.7% experienced severe dysfunction, 15.3% moderate, and 29.3% mild dysfunction. These findings align with previous research indicating that men with GWs are prone to sexual health challenges. For instance, a study by Nia et al. reported that 35.2% of men with GWs had overall sexual dysfunction, with the erectile domain being most affected (85.7%). This underscores the profound impact GWs can have on erectile function, a cornerstone of male sexual health [1].

Sexual dysfunction in men with GWs was notably prevalent in our study, with moderate dysfunction observed in erectile function (IIEF mean score: 14.3 ± 4.2), orgasmic satisfaction (5.8 ± 2.1), and overall satisfaction (6.5 ± 2.4). These findings align closely with those of Juang et al., whose nationwide cohort study on patients with HPV infection (*n* = 13,296) had a higher risk of subsequent ED in comparison to the non-HPV controls [11].

Several potential mechanisms may underlie the relationship between human papillomavirus (HPV) infection and erectile dysfunction (ED). Erectile function involves a complex neurovascular mechanism, dependent on both healthy blood flow and intact neural pathways. HPV infection may contribute to ED pathogenesis by inducing inflammation and accelerating the progression of atherosclerosis, a condition known to significantly impair vascular health and blood flow to erectile tissue [4].

Additionally, infectious agents—including viruses, bacteria, and parasites—can trigger autoimmune responses and have been linked to secondary vasculitis. In particular, HPV has been associated with granulomatous vasculitis in the genital area, which may further compromise vascular function and contribute to ED [5].

Interestingly, a recent study investigated the effects of GWs on men's depression and sexual functions. The study, which included 77 male patients and utilized the International Index of Erectile Function (IIEF-5) to assess erectile function, found that men with larger warts had significantly higher rates of erectile dysfunction. In their cohort, 52.5% of patients with larger lesions exhibited abnormal scores on the Hospital Anxiety and Depression Scale (HADS), indicating that psychological distress plays a critical role in sexual function outcomes. This contrasts with our findings, where only 10.7% of patients reported severe dysfunction [6]. The discrepancy may stem from differences in wart size, sample population characteristics, or the psychological assessment methods employed in each study [12]. Larger warts may cause greater distress, contributing to erectile dysfunction via anxiety-related mechanisms.

Other reports in align with our findings indicated that genital warts led to significant psycho-sexual distress, including feelings of stigma, social isolation, and diminished self-esteem. Many participants reported disruptions in their sexual lives, with some avoiding intimate relationships altogether due to the emotional burden associated with the condition [7, 13]. Conversely, Woodhall et al. found that patients reported relatively mild sexual function disruptions, based on assessments in multiple UK clinics [14]. However, their methodology differed, utilizing a broader psychological questionnaire rather than domain-specific tools like the IIEF or DLQI. This discrepancy suggests that generalized assessments may underreport the true burden of sexual dysfunction, underscoring the importance of validated, domain-specific tools for a more comprehensive evaluation.

### Quality of life implications

The Dermatology Life Quality Index (DLQI) scores in our study highlighted the significant burden GWs impose on daily life. A moderate impact was reported by 36.7% of participants, while 26.7% experienced a very large impact. These results are consistent with findings from Nia et al., who emphasized that sexual dysfunction in men with GWs significantly affects their sexual quality of life. Kucukunal et al. also reported that male patients with GWs have higher rates of sexual dysfunction, depression, and anxiety compared to the general population [2].This supports the growing body of evidence suggesting that both the physical and emotional burden of GWs contribute to impaired sexual function and overall well-being. The psychological distress stemming from GWs can lead to diminished self-esteem and strained interpersonal relationships, further exacerbating the negative impact on quality of life [15, 16].​

### Correlates of sexual dysfunction and quality of life

Our analysis identified several factors correlating with sexual dysfunction and diminished quality of life. Participants with warts persisting beyond six months exhibited higher rates of severe sexual dysfunction (20%) compared to those with shorter durations (5.3%). This suggests that prolonged exposure to the physical and psychological stressors associated with GWs can intensify sexual health issues.

Smokers demonstrated a higher prevalence of severe sexual dysfunction (7.3%) relative to non-smokers (3.3%). This aligns with broader literature indicating that smoking adversely affects vascular health, thereby impairing erectile function [17].​

Inconsistent or non-use of condoms was associated with greater quality-of-life impairments. Those who never used condoms reported higher rates of severe dysfunction (21.2%) compared to consistent users (6.7%). This may reflect heightened anxiety about transmitting or acquiring infections, further impacting sexual confidence and satisfaction [18].​ While higher educational level is typically associated with reduced risk of erectile dysfunction [19–21], our findings revealed an inverse relationship. This may reflect region-specific stressors, lifestyle patterns, or psychosocial burdens disproportionately affecting educated men in Egypt. It is also possible that increased awareness among this subgroup results in more accurate or self-critical reporting of sexual function impairments.

### Limitations of the study

While our study provides valuable insights, certain limitations warrant acknowledgment. The cross-sectional design precludes causal inferences. Additionally, factors such as the severity of GWs, partner status, and concurrent psychological conditions were not extensively explored. Future longitudinal studies incorporating these variables could offer more understanding of the interrelationships affecting sexual function and quality of life in men with GWs.​

## Conclusion

Genital warts significantly impair male sexual function and quality of life, with many patients experiencing moderate to severe erectile dysfunction and psychosocial distress. Our findings highlight the need for routine sexual health evaluations and psychosocial screening in affected individuals. Future research should explore longitudinal outcomes, assess the impact of treatment interventions on sexual and psychological domains, and include control populations to better establish causality.

## Supplementary Information

Supplementary Material 1.

## Data Availability

The data that support the findings of this study are available from the corresponding author upon reasonable request.

## Abbreviations

DLQI: Dermatology Life Quality Index
ED: Erectile Dysfunction
GW: Genital Wart
HPV: Human Papillomavirus
HADS: Hospital Anxiety and Depression Scale
IIEF: International Index of Erectile Function
IRB: Institutional Review Board
QoL: Quality of Life
SPSS: Statistical Package for the Social Sciences
