# Supplementary material for: Sexual dysfunction and quality of life in males with genital warts
Source: Basic Clin Androl. 2025 Jun 19;35:25. doi: 10.1186/s12610-025-00274-1 (PMC12177989; doi:10.1186/s12610-025-00274-1)
Supplement: Supplementary file 1 — Supplementary Material 1. [file 12610_2025_274_MOESM1_ESM.docx]

Supplementary table (1) Impact of Genital Warts on quality of life

| **DLQI Score Range** | **Impact on Life** | **n (%)** |
| --- | --- | --- |
| **0–1** | No impact | 8 (5.3) |
| **2–5** | Small impact | 35 (23.3) |
| **6–10** | Moderate impact | 55 (36.7) |
| **11–20** | Very large impact | 40 (26.7) |
| **21–30** | Extremely large impact | 12 (8.0) |

Legend: Describes quality of life impairment levels via DLQI score ranges. Patients grouped by severity. DLQI = Dermatology Life Quality Index.

Top of Form

Bottom of Form

Supplementary table (2) Prevalence of Sexual Dysfunction by Smoking Status

| **Smoking Status** | **IIEF Score Range** | **Category** | **n (%)** |
| --- | --- | --- | --- |
| **Non-Smoker** | 26–30 | No dysfunction | 5 (3.3) |
|  | 22–25 | Mild dysfunction | 10 (6.7) |
|  | 17–21 | Moderate-to-mild dysfunction | 12 (8.0) |
|  | 11–16 | Moderate dysfunction | 7 (4.7) |
|  | 0–10 | Severe dysfunction | 5 (3.3) |
| **Total for non-Smoker** |  |  | **39 (26.0)** |
| **Smoker** | 26–30 | No dysfunction | 26 (17.3) |
|  | 22–25 | Mild dysfunction | 26 (17.3) |
|  | 17–21 | Moderate-to-mild dysfunction | 32 (21.3) |
|  | 11–16 | Moderate dysfunction | 16 (10.7) |
|  | 0–10 | Severe dysfunction | 11 (7.3) |
| **Total for Smoker** |  |  | **111 (74.0)** |

Legend: Presents erectile dysfunction severity by smoking status. Data derived from IIEF categories. IIEF = International Index of Erectile Function.

Supplementary table (3) Correlation Between Duration of Warts and Sexual Function (IIEF Score)

| **Duration of Warts (months)** | **IIEF Score Range** | **Category** | **n (%)** |
| --- | --- | --- | --- |
| **≤ 6 months** | 26–30 | No dysfunction | 20 (21.1%) |
|  | 22–25 | Mild dysfunction | 28 (29.5%) |
|  | 17–21 | Moderate-to-mild dysfunction | 22 (23.2%) |
|  | 11–16 | Moderate dysfunction | 20 (21.1%) |
|  | 0–10 | Severe dysfunction | 5 (5.3%) |
| **Total for ≤ 6** |  |  | **95 (100%)** |
| **> 6 months** | 26–30 | No dysfunction | 11 (20.0%) |
|  | 22–25 | Mild dysfunction | 8 (14.5%) |
|  | 17–21 | Moderate-to-mild dysfunction | 22 (40.0%) |
|  | 11–16 | Moderate dysfunction | 3 (5.5%) |
|  | 0–10 | Severe dysfunction | 11 (20.0%) |
| **Total for > 6** |  |  | **55 (100%)** |

Legend: Shows sexual dysfunction levels by wart duration. Compares ≤6 months vs >6 months. IIEF = International Index of Erectile Function.

Supplementary table (4) Correlation Between Condom Use and Quality of Life (DLQI)

| **DLQI Score Range** | **Impact on Life** | **Always (n, %)** | **Sometimes (n, %)** | **Never (n, %)** | **p-value** |
| --- | --- | --- | --- | --- | --- |
| **0–1** | No impact | 5 (8.3%) | 2 (3.5%) | 1 (3.0%) | **0.03** |
| **2–5** | Small impact | 20 (33.3%) | 10 (17.5%) | 5 (15.2%) | **0.02** |
| **6–10** | Moderate impact | 20 (33.3%) | 20 (35.1%) | 15 (45.5%) | 0.06 |
| **11–20** | Very large impact | 10 (16.7%) | 20 (35.1%) | 10 (30.3%) | **0.04** |
| **21–30** | Extremely large impact | 5 (8.3%) | 5 (8.8%) | 2 (6.1%) | **0.05** |
| **Total** |  | **60 (100%)** | **57 (100%)** | **33 (100%)** |  |

Legend: Assesses impact of condom use on DLQI scores. Patients grouped by usage frequency. DLQI = Dermatology Life Quality Index.

Supplementary table (5) Correlation Between Condom Use and IIEF Score

| **IIEF Score Range** | **Category** | **Always (n, %)** | **Sometimes (n, %)** | **Never (n, %)** | **p-value** |
| --- | --- | --- | --- | --- | --- |
| **0–10** | (Severe dysfunction) | 4 (6.7%) | 5 (8.8%) | 7 (21.2%) | **0.02** |
| **11–16** | (Moderate dysfunction) | 9 (15.0%) | 11 (19.3%) | 13 (39.4%) | **0.01** |
| **17–21** | (Moderate-to-mild dysfunction) | 24 (40.0%) | 12 (21.1%) | 8 (24.2%) | **0.03** |
| **22–25** | (Mild dysfunction) | 12 (20.0%) | 11 (19.3%) | 3 (9.1%) | **0.04** |
| **26–30** | (No dysfunction) | 11 (18.3%) | 18 (31.6%) | 2 (6.1%) | **0.05** |
| **Total** |  | **60 (100%)** | **57 (100%)** | **33 (100%)** |  |

Legend: Displays erectile dysfunction severity by condom use. Statistically significant differences highlighted. IIEF = International Index of Erectile Function.

Supplementary table (6) Distribution of Sexual Dysfunction Categories by Age Group

| **Age Group (years)** | **18–24** | **25–29** | **30–39** | **≥40** | **Total** |
| --- | --- | --- | --- | --- | --- |
| **Severe Dysfunction (n)** | 5 | 6 | 3 | 2 | **16** |
| **Moderate Dysfunction (n)** | 4 | 10 | 6 | 3 | **23** |
| **Mild-Moderate Dysfunction (n)** | 14 | 16 | 9 | 5 | **44** |
| **Mild Dysfunction (n)** | 7 | 17 | 10 | 2 | **36** |
| **No Dysfunction (n)** | 5 | 11 | 12 | 3 | **31** |
| **Total (n)** | **35** | **60** | **40** | **15** | **150** |
| **p-value** | **0.03** | **0.05** | 0.09 | 0.07 |  |

Legend: Presents ED categories distributed across age groups. Shows p-values by age category. IIEF = International Index of Erectile Function.
